# Supplementary material for: Embryonic Deletion of TXNIP in GABAergic Neurons Enhanced Oxidative Stress in PV+ Interneurons in Primary Somatosensory Cortex of Aging Mice: Relevance to Schizophrenia
Source: Brain Sci. 2022 Oct 15;12(10):1395. doi: 10.3390/brainsci12101395 (PMC9599691; doi:10.3390/brainsci12101395)
Supplement: Supplementary file 1 [file brainsci-12-01395-s001.zip › Table S1.pdf]

Table S1. Demographical and clinical characteristics of the schizophrenia and healthy control group.

	Group				Analysis		
**Variable**	**FEDN (126)**		**HC (478)**		**Statistic**	***df***	***P***
	N	%	N	%			
**Sex**					$\chi^2 = 59.36$	1	< 0.0001
Male	85	67.2%	146	30.5%			
Female	41	32.8%	332	69.5%			
	Mean	SD	Mean	SD			
**Age (y)**	34.51	13.38	37.87	13.31	F = 6.56	1	P = 0.011
**Onset age (y)**	30.22	1.54					
**BMI (kg/m^2)**	21.50	3.18	22.14	2.96	F = 4.74	1	P = 0.030
**CPZ dose (mg/d)**	450.45	286.24					
**PANSS total score**	87.37	32.25					
P subscore	22.77	10.85					
N subscore	18.88	9.13					
G subscore	45.72	17.56					

Note:

BMI: Body Mass Index; CPZ, Chlorpromazine; PANSS, Positive and Negative Syndrome Scale; P, positive symptom; N, negative symptom; G, General psychopathology. FEDN: first-episode drug-naïve schizophrenia patients; HC: healthy controls
