# Supplementary material for: Embryonic Deletion of TXNIP in GABAergic Neurons Enhanced Oxidative Stress in PV+ Interneurons in Primary Somatosensory Cortex of Aging Mice: Relevance to Schizophrenia
Source: Brain Sci. 2022 Oct 15;12(10):1395. doi: 10.3390/brainsci12101395 (PMC9599691; doi:10.3390/brainsci12101395)
Supplement: Supplementary file 1 [file brainsci-12-01395-s001.zip › Table S2.pdf]

Table S2 Demographical and clinical characteristics of the schizophrenia subgroups and healthy control group.

	Group				Analysis		
**Variable**	**FEDN (⩾ 1.76ng/mL) （92）**		**HC (478)**		**Statistic**	***df***	***P***
	N	%	N	%			
**Sex**					$\chi^2 = 37.84$	1	P < 0.0001
Male	58	63%	146	30.5%			
Female	34	37%	332	69.5%			
	Mean	SD	Mean	SD			
**Age (y)**	33.85	13.43	37.87	13.31	F = 4.758	1	P = 0.030
**Onset age (y)**	30.17	13.40					
**BMI (kg/m²)**	21.61	3.20	22.14	2.96	F =1.936	1	P = 0.165
**CPZ dose (mg/d)**	421.60	252.14					
**PANSS total score**	90.91	32.56					
P subscore	24.43	11.33					
N subscore	18.99	9.52					
G subscore	47.49	17.94					

Note:

BMI: Body Mass Index; CPZ, Chlorpromazine; PANSS, Positive and Negative Syndrome Scale; P, positive symptom; N, negative symptom; G, General psychopathology. FEDN: first-episode drug-naïve schizophrenia patients; HC: healthy controls
