# Supplementary material for: Embryonic Deletion of TXNIP in GABAergic Neurons Enhanced Oxidative Stress in PV+ Interneurons in Primary Somatosensory Cortex of Aging Mice: Relevance to Schizophrenia
Source: Brain Sci. 2022 Oct 15;12(10):1395. doi: 10.3390/brainsci12101395 (PMC9599691; doi:10.3390/brainsci12101395)
Supplement: Supplementary file 1 [file brainsci-12-01395-s001.zip › Table S3.pdf]

Table S3 Demographical and clinical characteristics of the schizophrenia subgroups and healthy control group.

	Group				Analysis		
**Variable**	**FEDN (≤ 1.76ng/mL) （34）**		**HC (478)**		**Statistic**	***df***	***P***
	N	%	N	%			
**Sex**					$\chi^2 = 38.60$	1	P < 0.001
Male	28	82.4%	146	30.5%			
Female	6	17.6%	332	69.5%			
	Mean	SD	Mean	SD			
**Age (y)**	35.97	13.15	37.87	13.31	F = 0.656	1	P = 0.418
**Onset age (y)**	27.67	5.00					
**BMI (kg/m^2)**	21.24	3.17	22.14	2.96	F = 2.890	1	P = 0.090
**CPZ dose (mg/d)**	525.76	359.31					
**PANSS total score**	75.03	23.80					
P subscore	16.00	3.33					
N subscore	18.48	7.93					
G subscore	40.54	15.32					

Note:

BMI: Body Mass Index; CPZ, Chlorpromazine; PANSS, Positive and Negative Syndrome Scale; P, positive symptom; N, negative symptom; G, General psychopathology. FEDN: first-episode drug-naïve schizophrenia patients; HC: healthy controls
