# Supplementary material for: Embryonic Deletion of TXNIP in GABAergic Neurons Enhanced Oxidative Stress in PV+ Interneurons in Primary Somatosensory Cortex of Aging Mice: Relevance to Schizophrenia
Source: Brain Sci. 2022 Oct 15;12(10):1395. doi: 10.3390/brainsci12101395 (PMC9599691; doi:10.3390/brainsci12101395)
Supplement: Supplementary file 1 [file brainsci-12-01395-s001.zip › Table S4.pdf]

Table S4 Demographical and clinical characteristics of the schizophrenia subgroups.

	Group				Analysis		
**Variable**	**Group1 (⩾1.76 ng/mL) (92)[a]**		**Group2 (< 1.76 ng/mL) (34)**		**Statistic**	***df***	***P***
	N	%	N	%			
**Sex**					$\chi^2 = 4.272$	1	P = 0.039
Male	58	63%	28	82.4%			
Female	34	37%	6	17.6%			
	Mean	SD	Mean	SD			
**Age (y)**	33.85	13.43	35.97	13.15	F = 0.621	1	P = 0.432
**Onset age (y)**	30.17	13.40	27.67	5.00	F = 0.20	1	P = 0.652
**BMI (kg/m²)**	21.61	3.20	21.24	3.17	F = 0.31	1	P = 0.579
**CPZ dose (mg/d)**	421.60	252.14	525.76	359.31	F = 3.245	1	P = 0.074
**PANSS total score**	90.91	32.56	75.03	23.80	F = 6.52	1	P = 0.012
P subscore	24.43	11.33	16.00	3.33	F = 11.35	1	P < 0.0001
N subscore	18.99	9.52	18.48	7.93	F = 0.068	1	P = 0.794
G subscore	47.49	17.94	40.54	15.32	F = 3.915	1	P = 0.050

Note:

[a] *FEND patients were divided to two subgroups based on their TXNIP concentration in plasma.*

BMI: Body Mass Index; CPZ, Chlorpromazine; PANSS, Positive and Negative Syndrome Scale; P, positive symptom; N, negative symptom; G, General psychopathology. FEDN: first-episode drug-naïve schizophrenia patients; HC: healthy controls
